# Cerebral Vasospasm as a Delayed Complication Following Glioblastoma Resection

**DOI:** 10.1155/crnm/8872074

**Published:** 2025-11-14

**Authors:** Andrew C. Pickles, John T. Tsiang, Shiau-Sing Ciecierska, Ronak H. Jani, Joseph C. Serrone, Brandon J. Bond, Jigisha P. Thakkar, Vikram C. Prabhu

**Affiliations:** ^1^Stritch School of Medicine, Loyola University Chicago, Maywood, Illinois, USA; ^2^Department of Neurological Surgery, Loyola University Medical Center, Maywood, Illinois, USA; ^3^Department of Neurology, Loyola University Medical Center, Maywood, Illinois, USA; ^4^Department of Radiation Oncology, Loyola University Medical Center, Maywood, Illinois, USA

**Keywords:** cerebrovascular, endovascular, glioblastoma, neuro-oncology, vasospasm

## Abstract

Postoperative cerebral vasospasm is usually triggered by vasoactive metabolic blood products in the subarachnoid space but is rarely reported following resection of intrinsic diffuse lobar neoplasms such as malignant gliomas. This 34-year-old right-handed Caucasian lady underwent an uneventful resection of a right mesial temporal lobe glioblastoma with no postoperative neurological deficits. Eight days after her index surgery, she presented with left-sided hemiparesis and dysarthria and was found to have right M1 narrowing, consistent with cerebral vasospasm. Intra-arterial calcium channel blocker (CCB) administration and induced hypertension were started to treat the cerebral vasospasm and resulted in resolution of most of her neurological deficits. At 2 months postresection, she was noted to be without neurological deficits and able to proceed with appropriate adjuvant therapies for the glioblastoma. Postoperative cerebral vasospasm following resection of a glioblastoma can occur and present in a similar manner and timeframe as post-subarachnoid hemorrhage vasospasm. Prompt recognition of this condition followed by endovascular intervention and systemic treatments to improve cerebral perfusion are essential at reducing the risk of permanent cerebral ischemia and deficits.

## 1. Introduction

Glioblastoma multiforme (GBM) is the most common primary malignant brain tumor in adults [1]. The standard of care involves maximal safe resection followed by concurrent radiation and temozolomide (TMZ), maintenance TMZ, and tumor treatment fields as per the Stupp protocol [2–7]. Surgical resection of malignant gliomas usually targets the contrast-enhancing portion of the tumor, but there is growing evidence that maximal safe resection of the nonenhancing T2/FLAIR hyperintense component of the tumor may also have a salutary effect on overall survival [8, 9]. Surgical adjuncts such as the use of 5-ALA or sodium fluorescein are employed to aid with tumor visualization along with the use of intraoperative neuronavigation and ultrasound; some centers have access to intraoperative magnetic resonance imaging (MRI) as well [10]. These tumors can violate the pial boundaries and encase or involve major cerebral arterial vessels.

Cerebral vasospasm is most commonly encountered in clinical practice following spontaneous aneurysmal subarachnoid hemorrhage (SAH) and is postulated to be caused by the breakdown of vasoactive metabolic blood products in the subarachnoid space [11]. It has also been reported following resection of cranial base tumors, as these frequently extend into the basal cisterns involving the major arteries or their branches and perforators [12–15]. In these cases, factors associated with a higher likelihood of postresection cerebral vasospasm are reported to be vessel encasement, vessel narrowing, tumor size, hypothalamic dysregulation, and subarachnoid blood in the resection cavity [16–18]. Treatment for cerebral vasospasm and its sequelae initially involves administration of nimodipine, establishment of euvolemia, and systemic hypertensive therapy. Endovascular treatment includes intra-arterial (IA) calcium channel blockers (CCBs) and balloon angioplasty [19–22].

We report a case of cerebral vasospasm following resection of a mesial temporal lobe glioblastoma that presented in a manner similar to cerebral vasospasm following aneurysmal SAH and was treated successfully using similar principles as would be employed in the setting of post-SAH cerebral vasospasm. Our intent is to draw attention to this rare phenomenon that may complicate the recovery of a patient following glioblastoma resection and to provide an effective treatment algorithm that may mitigate any neurological deficits and facilitate the use of adjuvant therapies.

## 2. Case Presentation

**Figure 1:**
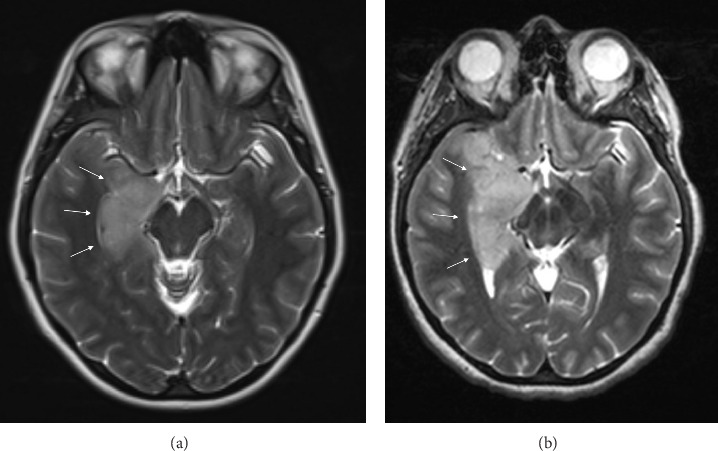
(a) Initial MRI head, T2-weighted sequence, demonstrating an area of hyperintensity (denoted by arrows) in the right mesial temporal lobe measuring 2.5 × 2.6 × 3.1 cm, and (b) 3-month follow up MRI head, T2-weighted sequence, demonstrating interval progression of mesial temporal lobe mass (again, denoted by arrows), now measuring 3.5 × 3.3 × 5.5 cm.

This 34-year-old female presented with a generalized tonic–clonic seizure and was found to have a right mesial temporal lobe T2/FLAIR hyperintense lesion (Figure 1(a)) without associated contrast enhancement. She was started on levetiracetam and referred to our facility. Three months later, her MRI revealed a significant increase in the area of T2/FLAIR hyperintensity (Figure 1(b)).

**Figure 2:**
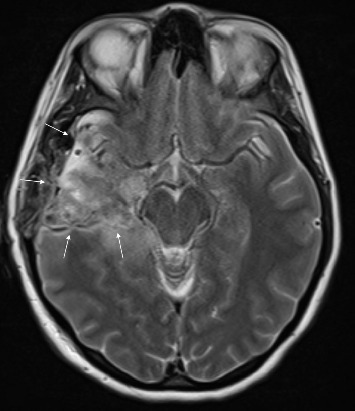
Postoperative day one MRI T2-weighted sequence, demonstrating extensive surgical resection (denoted by arrows) with a small T2 abnormality in the right hippocampus and surgical hemostatic product and a small amount of blood within the resection cavity.

She underwent a right temporal craniotomy and resection of the tumor. The surgical margins extended to the floor of the middle cranial fossa inferiorly, temporal pole anteriorly, and to the pia adjacent to the brainstem. In some areas, minor violations of the pia mater allowed blood products to enter into the basal cisterns and subarachnoid space. Copious saline irrigation was employed to wash out these blood products from the subarachnoid space and basal cisterns. The right superior cerebellar artery, posterior cerebral artery, and middle cerebral artery (MCA) were visualized and carefully protected with cotton patties. At the conclusion of the operation, the resection bed was gently lined with hemostatic product, the bone flap was replaced, and the scalp was closed appropriately. No intraoperative complications were encountered during the procedure. The patient awoke from anesthesia without any apparent deficits, and a routine postoperative MRI demonstrated a small residual T2 abnormality in the right posterior hippocampus (Figure 2).

The patient was discharged home 4 days after index surgery after an uneventful hospital stay. Pathology demonstrated an IDH wild-type, MGMT unmethylated GBM.

**Figure 3:**
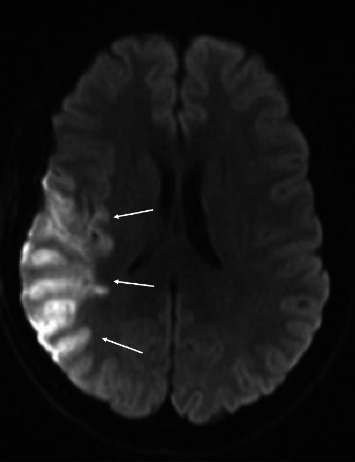
MRI head, diffusion-weighted imaging, demonstrating diffusion restriction along the right MCA territory (denoted by arrows).

Eight days after surgery, the patient presented with a sudden onset of left facial droop, left sided hemiparesis, and dysarthria. A CT angiogram demonstrated right M1 narrowing with decreased distal MCA opacification along the right parietal lobe. No large vessel occlusion (LVO) was noted. An MRI revealed diffusion restriction of the right MCA territory (Figure 3).

**Figure 4:**
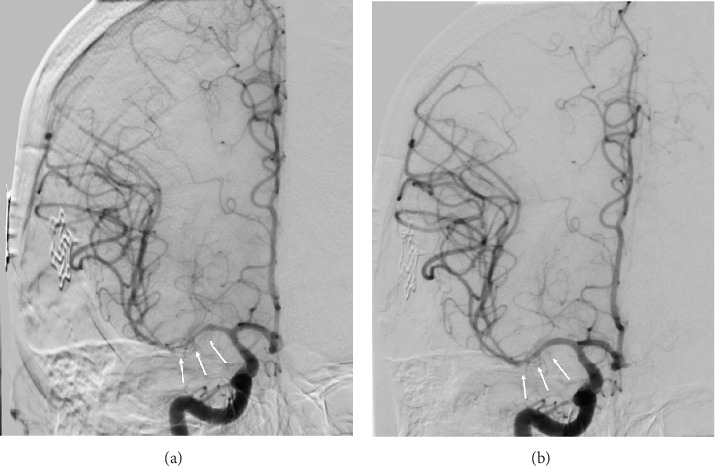
(a) DCA of right ICA before vasospasm treatment with IA vasodilators. Arrows denote vasospasm seen within the right M1 segment. (b) DCA of right ICA after vasospasm treatment with IA vasodilators. Arrows denote improvement seen in vessel caliber of the right M1 segment.

The patient's left-sided hemiparesis then self-resolved, with only a trace left upper extremity drift and left sided facial weakness remaining. Given the suspicion for vasospasm, the patient was allowed permissive hypertension to a systolic blood pressure < 180 mm Hg. A diagnostic cerebral angiogram (DCA) was performed on postoperative day (POD) nine noting severe vasospasm noted in the right M1 and proximal M2 (Figure 4(a)). 20 mg of IA verapamil and 10 mg IA nicardipine were injected in the right internal carotid artery (ICA), resulting in significant improvement in vessel caliber (Figure 4(b)).

After the procedure, her systolic blood pressure was maintained between 140–150 mm Hg for the duration of her hospital admission with norepinephrine. The patient was also started on oral nimodipine 60 mg every 4 hours for 7 days to promote vessel patency. Bedside transcranial Doppler (TCD) ultrasound obtained the next day demonstrated elevated Lindegaard ratios [23], suggesting that the patient was still in vasospasm, and she was taken back to the endovascular suite for a repeat DCA. The patient was again noted to have severe vasospasm of the right M1, and 20 mg IA verapamil and 10 mg IA nicardipine were administered to the right ICA with restoration of vessel caliber. Continued TCD velocity elevations suggested persistent vasospasm but the patient refused further treatment after the second intervention. Her neurological exam steadily improved and she was discharged home 10 days after her index admission.

**Figure 5:**
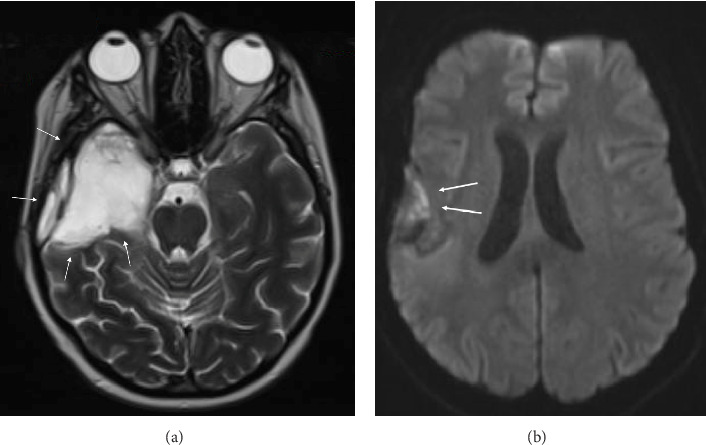
Six-month follow-up MRI head, demonstrating (a) no evidence of recurrence of the lesion on T2 sequence (denoted by arrows) and (b) improvement of diffusion restriction along the right MCA territory with minimal remaining signal (again, denoted by arrows).

At 2-week follow-up, the patient had full strength in her left hemibody with subtle left-sided facial weakness. She was started on the Stupp protocol for treatment of her GBM 1 month after her initial resection. At 3 months postresection, she had no focal neurologic deficits and was tolerating chemoradiation treatment well. MRI obtained demonstrated no recurrence of the lesion on both T1-weighted postcontrast and T2-weighted sequences (Figure 5(a)) and resolution of DWI abnormalities (Figure 5(b)).

## 3. Discussion

Although postresection perilesional ischemia is frequently noted following a malignant glioma resection, cerebral vasospasm is rare. Prior anecdotal reports suggest peri-Sylvian tumor resections may be at higher risk for vasospasm, particularly if there is surgical entry into the basal cisterns and Sylvian fissure, or contamination of these regions with blood products [24]. This may have been the pathophysiology of the vasospasm noted in our case. Research from SAH-induced vasospasm suggests that the breakdown of blood products in the subarachnoid space may produce vasoactive metabolites that activate calcium-dependent and independent channels that contribute to vasospasm [11].

A PubMed search was performed using the phrases “Glioma AND Vasospasm” and “Glioblastoma AND Vasospasm” on November 20, 2024. A total of 29 articles were identified. Twenty-six articles were excluded because they did not contain subject matter relevant to GBM resection complicated by cerebral vasospasm or were not available in English text. One article was excluded due to postoperative *Streptococcus oralis* meningitis preceding the development of vasospasm after GBM resection [25]. Two articles were included in our analysis detailing two patients with GBM resection complicated by cerebral vasospasm (Table 1).

In our review of the literature and present case, two of the three patients were female, and the mean age was 49.9 years (range 35–63). All three cases involved GBMs of the temporal lobe with two tumors also involving the insula. In the two cases reported in the prior literature, there was no description of blood in the basal cisterns. All three cases noted expected postoperative brain edema on routine imaging. In the two cases reported in prior literature, the surgeons had placed carmustine wafers in the resection cavity. Carmustine wafers were not used in our present case. In all three cases, vessels affected by vasospasm were noted to either be displaced or encased by the GBM; however, no direct vessel injury was noted during any of the procedures. Average time to symptomatic vasospasm was 9.3 days postoperatively (range 8–12 days). Two of the three cases were treated in the endovascular suite with IA CCBs, and in three cases, nimodipine was continued for several days postangiography. One case of vasospasm resulted in permanent left upper extremity plegia and left lower extremity paresis, while another resulted in severe hemiparesis even after rehabilitation. Our case demonstrated recovery with minimal persistent deficits.

Placement of carmustine wafers (Gliadel, Azurity Pharmaceuticals, Inc., Woburn, MA, USA) in the resection cavity is also purported to contribute to arterial vasospasm. However, they were not employed in our case, and we have not experienced vasospasm in other cases in which these wafers were deployed in the resection cavity. One theory proffered is that carmustine has vasoactive properties that may be responsible for vasospasm, especially in instances where large cerebral vessels are in close proximity to the resection cavity [21, 26]. Cerebral edema and a cytotoxic inflammatory marker release may also contribute to vasospasm. Cerebral vasospasm following skull base tumor resection is described in approximately 2% of patients generally associated with meningioma resections [15]. However, arterial vasospasm has also been reported following pituitary adenoma resections, and more rarely vestibular schwannomas [22, 27], particularly in patients with thick subarachnoid blood or intraparenchymal hemorrhage following surgery. Larger lesions with parasellar extension are at an even higher risk for vasospasm following surgical resection.

Similar to post-SAH vasospasm, the average time of onset of symptoms related to vasospasm following glioma resection appears to be 8–12 days following surgery, and the clinical deficits reflect the arterial territory involved. Hence, once other possible etiologies for a new-onset neurological deficit > 1 week out from surgery, such as seizures, infection, or hematoma formation, have been ruled out with appropriate imaging studies and electroencephalography, it is advisable to evaluate the cerebral vasculature for possible vasospasm. TCD, cerebrovascular perfusion studies (i.e., CT perfusion), CT or MR angiography, and catheter angiography of the cerebral vessels allow recognition of vasospasm [28–30]. Initial imaging should follow clinical stroke guidelines, by first employing a noncontrast CT scan to rule out hemorrhage, followed by a CT perfusion scan to identify areas with reduced blood flow, and CT angiography to rule out LVO [28]. If CT angiography is equivocal, MR angiography may be employed due to its greater sensitivity detecting subtle ischemic changes and identifying the ischemic core [29]. CT and MR perfusion studies are especially useful for identifying penumbra [30], or salvageable tissue surrounding the irreversibly damaged ischemic core, the extent of which may help guide treatment and prognostic discussions. TCD is a noninvasive means of monitoring for vasospasm and response to therapies [23, 31]; TCD velocities have a sensitivity and specificity of 69.2% and 84.4%, respectively, and by Lindegaard ratios, the sensitivity and specificity are 76.9% and 87.4%, respectively [23, 31]. However, while TCDs can be used to determine resolution of vasospasm, they should be supplemented by careful clinical examinations and cerebral perfusion studies or catheter angiography, if needed. In all three cases, there were focal vessel vasospasms with localizing symptoms. There were no reports of distal or diffuse vasospasm, which can most often be observed after SAH [11].

It is important for clinicians to recognize the signs of postoperative vasospasm, however rare the clinical entity may be. Prompt recognition allows for early institution of therapies; IA CCBs supplemented by intravenous or oral CCBs were effective in the case reported here. A delay in recognition of cerebral vasospasm and timely institution of vasodilatory therapies may result in permanent deficits that further complicate and negatively impact adjuvant therapies for the malignant glioma [32–35]. Endovascular DCA with intervention allows recognition of the vasospasm and administration of IA CCBs; in some instances, angioplasty of the vasospastic vessel may also be performed [36]. Systemic vasodilating CCBs may also be administered [36]. In our case, oral nimodipine was only administered for 7 days due to issues with hypotension following administration, symptomatic improvement, and a desire to wean the patient off vasopressors prior to discharge. However, the typical duration of therapy is 60 mg every 4 h for 21 days, based on recommendations for reducing vasospasm in SAH after ruptured cerebral aneurysms [37].

This represents a hybrid approach to treatment that combines systemic treatment traditionally used for SAH-induced vasospasm, with endovascular interventions targeted toward restoring focal flow deficits. To prophylactically treat vasospasm, some providers are borrowing techniques from aneurysm surgeries, including irrigating intraoperatively with dilute vasodilator, or lining the resection cavities with papaverine-soaked gel foam [38]. It is unclear at this time the efficacy of these techniques. Postoperative cerebral vasospasm may follow uncomplicated surgical resection of a malignant glioma. The timeline of clinical presentation and the radiographic studies reveal a similar semiology as noted with post-SAH vasospasm. Prompt recognition and treatment of symptomatic vasospasm is essential to restore vessel caliber and prevent irreversible delayed cerebral ischemia, morbidity, and/or mortality and also allows resumption or continuation of adjuvant therapies for the primary brain tumor.

## Figures and Tables

**Table 1:** Literature review of previously reported cases of glioblastoma resection complicated by cerebral vasospasm

First author, year	PMID	Age, sex	Tumor presentation	Tumor location	Vessels involved	Surgery	Vasospasm presentation	Vasospasm vessel(s)	Time to vasospasm	Vasospasm treatment	Sequelae
Nakada et al., 2015 [19]	28663960	63, male	Not described	L temporal lobe and insula	L lenticulostriate artery	L frontotemporal craniotomy	AMS and R hemiplegia	L lenticulostriate artery	12 days	Ozagrel sodium and edaravone for 2 weeks	L hemiparesis
Khan et al., 2020 [21]	32637221	51, female	Headaches	R temporal lobe and insula	Sylvian vessels	R frontotemporal craniotomy	Dysarthria, L facial weakness, L hemiplegia	R supraclinoid ICA, carotid terminus, R A1, R M1	8 days	IA nicardipine, angioplasty of R MCA and R supraclinoid ICA; permissive hypertension, oral nimodipine	Resolution of dysarthria, but persistent LUE plegia and LLE paresis
Present case	N/A	34, female	Seizure	R mesial temporal lobe	R MCA	R temporal craniotomy	L facial droop, L sided hemiparesis, dysarthria.	R M1, R M2	8 days	IA verapamil, IA nicardipine, SBP 140-150, nimodipine	Neurologically intact

*Note:* A1 = first segment of anterior cerebral artery; L = left; M1 = 1st segment of middle cerebral artery; M2 = 2nd segment of middle cerebral artery; R = right.

Abbreviations: AMS, altered mental status; IA, intra-arterial; ICA, internal carotid artery; LLE, left lower extremity; LUE, left upper extremity; MCA, middle cerebral artery; SBP, systolic blood pressure.

## Data Availability

The data that support the findings of this study are available from the corresponding author upon reasonable request.
